# Supplementary figures and images for: Deep learning identifies histopathologic changes in bladder cancers associated with smoke exposure status
Source: PLoS One. 2024 Jul 31;19(7):e0305135. doi: 10.1371/journal.pone.0305135 (PMC11290674; doi:10.1371/journal.pone.0305135)

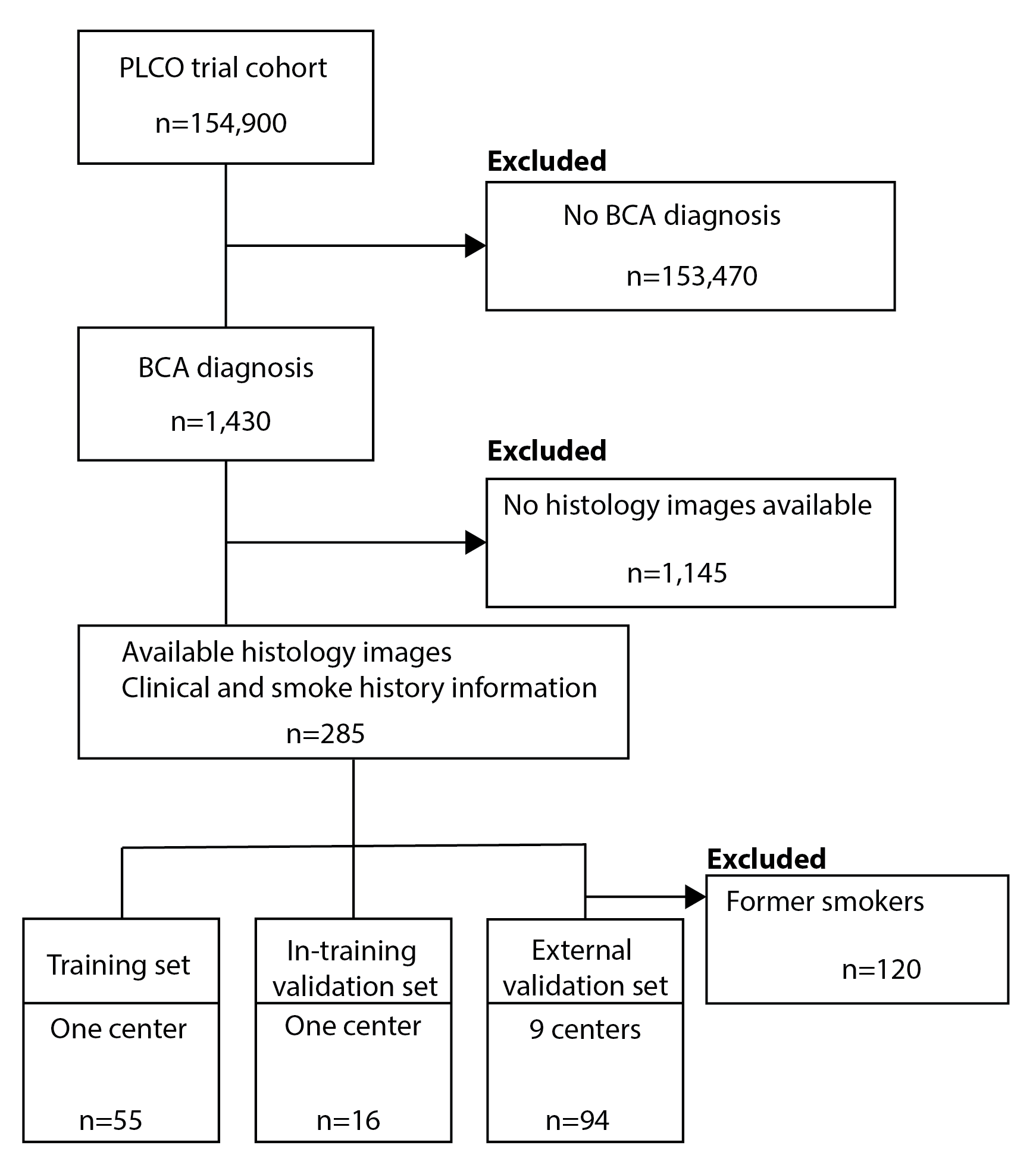

Supplement: S1 Fig — (PNG) [file pone.0305135.s001.png]

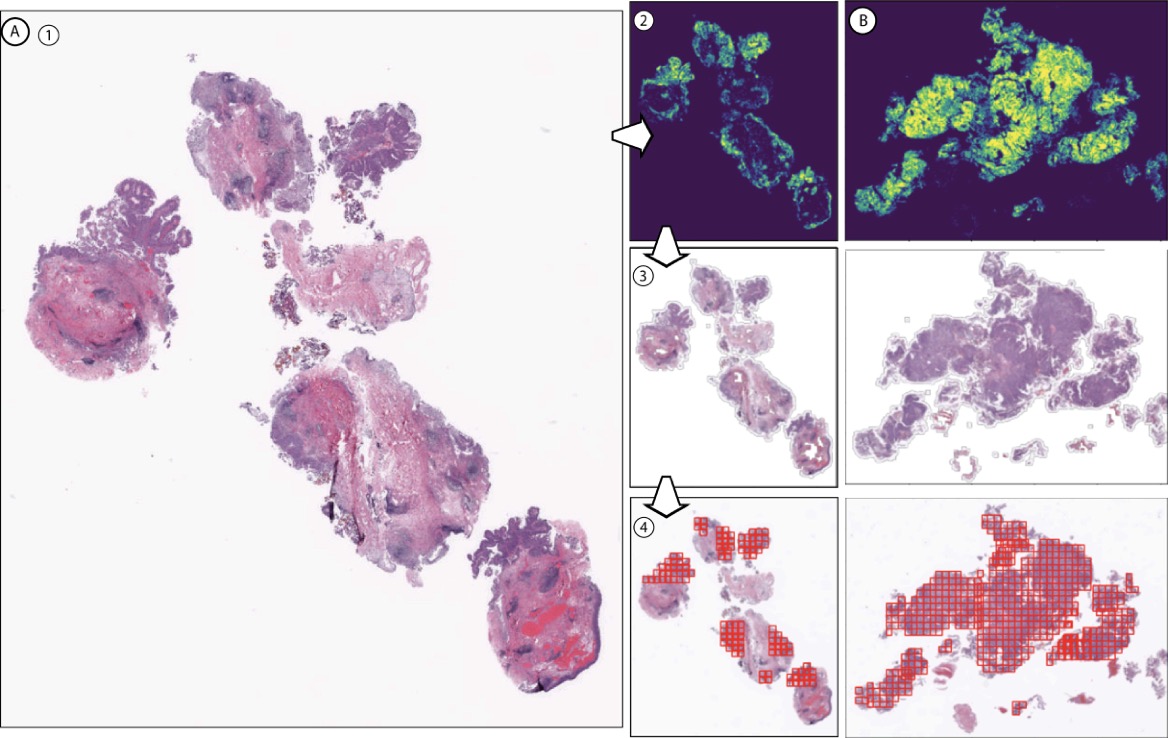

Supplement: S2 Fig — (A) a whole slide image (WSI) with bladder tissue samples; (1) the figure shows the distinctive coloration of hematoxylin-stained nuclei and contrasting appearance of urothelium from less cellular stromal tissue. (2) we applied a color mask to the WSI thumbnails, whose pixels have hue, saturation, and value (HSV) colors located in the color range between (H:140, S:20, V:50) and (H:150, S:255, V:255); (3) Background noise was then filtered from the WSI using the erosion function (kernel size: 2 x 2). Masked areas were expanded, and missing portions were filled with the dilation function (kernel size: 5 x 5); (4) masked areas were split into 10% overlapping squares and subsequently rescaled and remapped to the original WSI size. The masked areas were tiled on WSI at 10x magnification into small patches (512x512 pixels, one pixel = 1 μm) for processing due to memory constraints. (B) illustrates the processing steps on another whole slide image. (JPEG) [file pone.0305135.s002.jpeg]

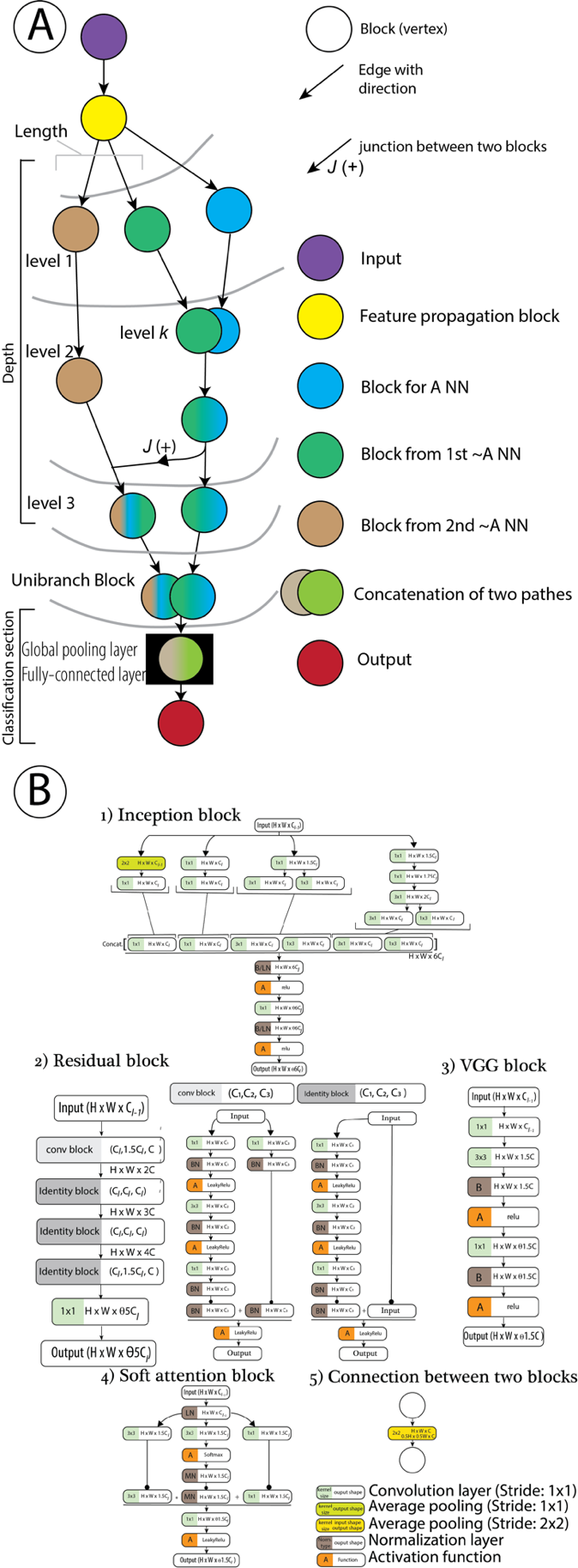

Supplement: S3 Fig — PlexusNET Architecture configuration is determined by depth, width, junctions, and blocks; (B) Blocks are composed of various neural network layers. MN: min-max normalization [–1,1], LN: layer normalization. BN: batch normalization. NN: neural networks. (PNG) [file pone.0305135.s003.png]
