# Supplementary material for: Deep learning identifies histopathologic changes in bladder cancers associated with smoke exposure status
Source: PLoS One. 2024 Jul 31;19(7):e0305135. doi: 10.1371/journal.pone.0305135 (PMC11290674; doi:10.1371/journal.pone.0305135)
Supplement: S1 Table — (DOCX) [file pone.0305135.s005.docx]

# **Supplementary Table 1**

Supplementary table 1 lists the overall impression and observation made by two senior uro-pathologists for all subspaces.

|  | Subspace 1 (Never smoker) | | Subspace 2 (Active smoker) | | Subspace 3 (Active smoker) | |
| --- | --- | --- | --- | --- | --- | --- |
|  | Pathologist 1 | Pathologist 2 | Pathologist 1 | Pathologist 2 | Pathologist 1 | Pathologist 2 |
| Overall impression | Dominance of moderate to poorly differentiated (G2-G3, high-grade) pTa urothelial cancers. | Dominance of high-grade urothelial carcinoma | indeterminant dominance (mixture of benign, lower, and high-grade cancers) | Dominance of high-grade urothelial carcinoma | Dominance of moderate-to-well differentiated (G1-G2) low-grade pTa urothelial carcinoma | Dominance of low-grade papillary urothelial carcinoma |
| Additional findings | well-differentiated (G1-G2) low-grade pTa urothelial cancers  poorly differentiated (G3) high-grade pT1 urothelial cancers  high-grade carcinoma-in-situ  Papillary urocystitis | high-grade urothelial carcinoma in different stages.  Artifacts resulting from slide preparation | Squamous metaplasia  Urocystitis with papillary hyperplasia  well-differentiated papillary Ta urothelial carcinoma (low-grade G1-2)  poorly differentiated Ta urothelial carcinoma (high-grade, G3) | Squamous metaplasia  Microcystic and glandular features in bladder cancers or as form of urocystitis  Low-grade looking tumors with prominent umbrella cell atypia  high-grade urothelial carcinoma  Artifacts due to cauterization | Mostly, but all can be low-grade urothelial carcinomas | Mostly low-grade, with few high-grade urothelial carcinomas. |
| Heterogeneity | heterogenous | heterogenous | heterogenous | heterogenous | homogenous | homogenous |
