# Supplementary material for: Deep learning identifies histopathologic changes in bladder cancers associated with smoke exposure status
Source: PLoS One. 2024 Jul 31;19(7):e0305135. doi: 10.1371/journal.pone.0305135 (PMC11290674; doi:10.1371/journal.pone.0305135)
Supplement: S1 File — (DOCX) [file pone.0305135.s006.docx]

Additional material for reviewers

*Deep Learning Identifies Histopathologic Changes in Bladder Cancers associated with Smoke Exposure Status*

Online access to the figures representing subspace 1 to 3.

Notice: These images are large, and the viewer tools provided by the operating system.

may not open them.

Please first download them and open them either with QuPath (https://qupath.github.io ) or Adobe Photoshop (A trial version can be downloaded from https://www.adobe.com/products/photoshop.html) .

Subspace 1 (Never smoker): <https://drive.google.com/file/d/1jpQiARWrIyCMTfo7QCoWmCAzaRWSbNV4/view?usp=sharing>

Subspace 2 (Active smoker):

<https://drive.google.com/file/d/1cf9HW-AHzmZYc58d36AMfXf2Y0USxTXe/view?usp=sharing>

Subspace 3 (Active smoker):

<https://drive.google.com/file/d/1yCH0v6tUATijVjNsyyJDaFafJdRe4dt_/view?usp=sharing>
