# Supplementary material for: Deep learning identifies histopathologic changes in bladder cancers associated with smoke exposure status
Source: PLoS One. 2024 Jul 31;19(7):e0305135. doi: 10.1371/journal.pone.0305135 (PMC11290674; doi:10.1371/journal.pone.0305135)
Supplement: Attachment — Submitted filename: Review.docx [file pone.0305135.s007.docx]

We would like to express our gratitude to the reviewer for their valuable comments. Based on their feedback, we have made the following edits to the text:

***Reviewer #1:***

*Summary
This paper proposed to predict smoke exposure status with histologic changes of BC. A total of 483 whole slide histology images (WSI) of 285 unique cases of BC collected from multiple centers for BC diagnosis were used in the study. A deep learning model was developed to predict the smoke exposure status and externally validated on BC cases. The model achieved an AUC of 0.67in the validation set.*

*Weakness
1. The AUC(0.64 with continuous parameter and 0.67 with categorized parameter) is rather low in the validation set. Better than random guess is not sufficient enough to draw the conclusion that histopathologic features are predictive for smoke exposure status.*

Author’s reply:

We appreciate the reviewer for raising this point. According to established statistical conventions [1-5], our analyses possess a robust statistical power of 82.2% at the case level, surpassing the widely accepted threshold of 80% at an alpha level of 0.05. This indicates that the likelihood of our hypothesis being a false positive is only 5%. Therefore, the robustness of our study allows us to derive the conclusion that histopathologic features are likely associated with smoke exposure status. Surely, the accuracy is subject to improvement in the future work. In multivariate mixed effect analyses, we found that the exposure score is the only independent predictor for the active exposure in contrast to BC grade.

We added the following sentence in Statistical analysis section, page 11:

“The statistical robustness of AUC was estimated using the statistical Power. We set the decision threshold for a robust statistical Power to 80% at an alpha level 0.05 (27-31).”

We added the following sentence in the result section, page 13:

“The statistical power of the study was determined to be 82.2%, surpassing the commonly accepted threshold of 80% at an alpha level of 0.05.”

*2. The authors need a much larger dataset for this study. A total of 367 WSI from 214 cases is sufficient for developing models for cancer subtypes classification or survival analysis. However, much more evidence and more convincing results are needed to reveal the correlation between histologic changes and smoke exposure. Hence a much larger dataset is necessary.*

Author’s reply:

We thank the reviewer once again for bringing up this point. According to our power analyses, we do not see any evidence to support the need for a much larger dataset. In our case, the inclusion of larger datasets may unnecessarily overpower the study and result in highlighting minor differences as significant, according to the common statistical conventions [1-5].

[1] Bacchetti P. Current sample size conventions: flaws, harms, and alternatives. BMC medicine. 2010;8:1-7.

[2] di Stephano J. How much power is enough? Against the development of an arbitrary convention for statistical power calculations. Functional Ecology. 2003;17:707-9.

[3] Greenland S, Senn SJ, Rothman KJ, Carlin JB, Poole C, Goodman SN, et al. Statistical tests, P values, confidence intervals, and power: a guide to misinterpretations. European journal of epidemiology. 2016;31:337-50.

[4] Lipsey MW. Design sensitivity: Statistical power for experimental research: sage; 1990.

[5] Myors B, Murphy KR, Wolach A. Statistical power analysis: A simple and general model for traditional and modern hypothesis tests: Routledge; 2014.

*3. The model is developed under single magnification (10x magnification with a patch size of 512x512 pixels). Why is the model able to capture histologic changes under 10x
magnification? What's the performance under different magnifications(5x, 20x)? I'd like to
see a detailed discussion about the impact of magnification selection on smoke exposure
status prediction.*

Author’s reply:

We trained a PlexusNET model on 20x magnification. However, we observed performance degradation on the test set (AUROC: 0.528; 95% CI: 0.432 – 0.624 according to DeLong), indicating that magnification does impact performance. Importantly, the association between histology images and smoke exposure status at 20x magnification is random, whereas the association at 10x magnification is significant and not random. This finding indicates that histologic patterns (i.e., tissue architecture) at 10x magnification are predictive of smoke exposure, supporting the key message of the current work regarding the potential association between active smoke exposure and histological images.

We added the following paragraph in the method section, page 8:

“To investigate the effect of the magnification, we repeated the previous steps (except the NAS step; here, we used the resulting model architecture from the first evaluation) to develop and validate a prediction model on images at 20x magnification. After fixing the optimal magnification level, we also examined the attention-based multi-instance learning defined according to Ilse et al. [6] as it has shown performance benefits for certain tasks in digital pathology [7, 8]. The base model was the PlexusNet model determined by NAS that will be described in the following section.”

We added the following paragraph in the result section, page 15:

“When comparing models trained on histology images at 10x magnification versus those trained on images at 20x magnification, we observed performance degradation on the external validation set (AUROC: 0.528; 95% CI: 0.433 – 0.624), indicating that magnification does impact performance.”

We added the following paragraph in the discussion section, page 21:

“The association between histology images and smoke exposure status at 20x magnification is random, whereas the association at 10x magnification is significant and not random. This finding indicates that histologic patterns (i.e., tissue architecture) at 10x magnification are predictive of smoke exposure, supporting the key message of the current work regarding the potential association between active smoke exposure and histological images.“

*4. The authors used a CNN architecture for smoke exposure prediction. However, multiple instance learning (MIL) is more common for WSI analysis[1,2,3], because CNNs tend to capture local features of WSIs while MILs can represent the global features of WSI. Additional experiments of MIL and a discussion about the impact of local features and global features on smoke exposure prediction are recommended for the study.*

*Many papers show the sufficiency of MIL in solving prediction problems.*

[1] Campanella, Gabriele, Matthew G. Hanna, Luke Geneslaw, Allen Miraflor, Vitor Werneck Krauss
Silva, Klaus J. Busam, Edi Brogi, Victor E. Reuter, David S. Klimstra, and Thomas J. Fuchs. "Clinicalgrade computational pathology using weakly supervised deep learning on whole slide images."
Nature medicine 25, no. 8 (2019): 1301-1309.
[2] Lu, Ming Y., Drew FK Williamson, Tiffany Y. Chen, Richard J. Chen, Matteo Barbieri, and Faisal
Mahmood. "Data-efficient and weakly supervised computational pathology on whole-slide
images." Nature biomedical engineering 5, no. 6 (2021): 555-570.
[3] Yu, Jin-Gang, Zihao Wu, Yu Ming, Shule Deng, Yuanqing Li, Caifeng Ou, Chunjiang He, Baiye
Wang, Pusheng Zhang, and Yu Wang. "Prototypical multiple instance learning for predicting lymph
node metastasis of breast cancer from whole-slide pathological images." Medical Image Analysis
(2023): 102748.

Author’s reply:

As requested, we considered an Attention-based Multiple Instance Learning (MIL) approach for the model at 10x magnification. However, we did not find performance benefits compared to the non-MIL approach for our research question, indicating the need to customize the model design according to the study question and the absence of a universal solution. Furthermore, the prediction of the MIL approach for smoke status is likely random based on the 95% Confidence Interval for AUROC, which was between 0.437 and 0.685.

In the current study, we further investigated the local features using 3D t-SNE and visualized the 3D feature spaces. We identified global feature subspaces that revealed a high concentration for patches dominated by either active smokers or non-smokers. These findings further indicate reorganization of histologic features associated with these subspaces. Our pathologists investigated and confirmed distinguishable histologic patterns between the subspaces we identified using 3D t-SNE visualization. We emphasize that the 3D visualization of feature spaces provides significantly more information than the 2D visualization of the feature spaces.

We added the following paragraph in the method section, pages 8-9 :

“After fixing the optimal magnification level, we also examined the attention-based multi-instance learning defined according to Ilse et al. [6] as it has shown performance benefits for certain tasks in digital pathology [7, 8]. The backbone model was the PlexusNet model we defined earlier.”

We added the following paragraph in the result section, page 15:

“In addition to that, MIL failed to provide a non-random prediction for the smoke exposure status (AUROC: 0.561; 95% CI: 0.437 – 0.685).”

We added the following paragraph in the discussion section, pages 21 and 22:

“In the current study, we further investigated the local features using 3D t-SNE and visualized the 3D feature spaces. We identified global feature subspaces that revealed a high concentration for patches dominated by either active smokers or non-smokers. These findings further indicate reorganization of histologic features associated with these subspaces. Our pathologists investigated and confirmed distinguishable histologic patterns between the subspaces we identified using 3D t-SNE visualization. We emphasize that the 3D visualization of feature spaces provides significantly more information than the 2D visualization of the feature spaces.”

“According to the evaluation of the MIL approach, we did not find performance benefits compared to the non-MIL approach for our research question, indicating the need to customize the model design according to the study question and the absence of a universal solution. Furthermore, the prediction of the MIL approach for smoke status is random based on the 95% Confidence Interval for AUROC, which was between 0.437 and 0.685.”

**Reviewer #2:**

*This article use the deep learning model of previous published PlexusNet to distinguish never smoker vs active smoker by analysis patches of whole slice histology images on the PLCO cancer screening trial.

1. You stated about refs 6~11, “these data are overall limited.” Please explain them in detail. And state your novelty.*

Author’s reply:

We do not know whether histologic appearance in histology images is associated with smoke exposure. The current work is the first study to investigate this association.

We modified the following section in the introduction, page 3:

“However, the existing data from previous studies are characterized by overall inconsistencies and a focus on the controversial association between BC grade and smoke exposure. As a result, it remains unclear whether there is indeed an association between specific histologic features in BC and smoke exposure. Given this knowledge gap, the current study investigated the potential association between histology images and smoke exposure status.”

*2. Please add a purpose section in abstract.*

Author’s reply:

Added accordingly in the abstract.

“Given this knowledge gap, the current study investigated the potential association between histology images and smoke exposure status.”

*3. Abstract Results section: “non-randomness” ,why you would like to use “non-randomness”? Do you imply your model is better than a random classifier? I suggest you use another model to do comparison instead stating yours are better than randomness.*

Author’s reply:

In statistical terms, "non-randomness" refers to the presence of significant non-random associations between histology and active smoke exposure status. The main aim of this work is to reveal the association between histology images and smoke exposure using deep learning. We also used BC grade as a reference variable, which was not associated with smoke exposure, in contrast to our smoke score.

*4. Can you draw a figure of data flowchart with inclusion and tons exclusion from 154,900 participants to 1430 BC to 285 cases of development, optimization, and external validation.*

Authors’ reply:

We appreciate the suggestion to provide a flowchart illustrating the data flow. We have included a supplementary figure in the paper to illustrate the process, showing the inclusion and exclusion of participants from 154,900 to 1,430 breast cancer cases, and further narrowing down to 285 cases for development, optimization, and external validation.

*5. Please use consistent terms of development/training, optimization/in-training validation through the paper.*

Authors’ reply:

We have revised the text to ensure consistent use of terms such as training and optimization/in-training validation throughout the paper. We used the term development set to covers both the training set and the in-training validation/optimization set.

*6, Excluding former smokers from external validation doesn’t make sense. You’re trained and validate your model with three categories including former smokers. Please write the details about how did you transfer three category outputs to two category outputs.*

Authors’ reply:

We disregarded the classification prediction for former smokers and only considered the prediction score for active smokers for the association analyses. The primary reason for including the class for former smokers during model development is to regularize the model prediction. However, considering the former smoker status for external validation is not appropriate given the difficulty in identifying the regeneration level of the tissues resulting from stopping smoke exposure. In contrast, we can assume that the baseline environmental exposure (including passive smoking) affects both smokers and non-smokers equally, while smokers have direct exposure to tobacco.

*Accordingly, we added the previous paragraph in the discussion section, pages 20 and 21.*

*7. Please draw your deep learning network architecture.*

Authors’ reply:

We appreciate the reviewer's request to provide the model architecture. However, instead of drawing the network architecture, we utilized a recently published model architecture concept that emphasizes the complexity of visualization. We believe this approach will be more beneficial for readers to understand the model's structure and functionality.

*8. Why did you just use grid search instead of Bayesian Optimization, Hyerband, or random search.*

Authors’ reply:

The search space for architecture hyperparameters is finite, limited, and searchable using the conventional grid search. The computation cost for grid search is also reasonable.

Accordingly, we added the following sentences in the Method section, page 7:

“The grid search was preferred because the search space for architecture hyperparameters was finite, limited, and searchable using the conventional grid search. The computation cost for grid search was also reasonable.”

*9. There is an extra space between “age” and “at diagnosis” in the equation of the mixed effect regression.*

Authors’ reply:

Corrected accordingly.

*10. Please explain why the mixed effect regression equation use exposure-score * cancer-grade instead of exposure-score + cancer-grade.*

Authors’ reply:

Both parameters are derived from histology appearance, and we anticipated the existence of interaction between them. From statistical perspective, we applied this to achieve a non-singular fit in contrast to *exposure-score + cancer-grade that resulted in a singular fit when the random effect including gender and age at diagnosis is considered.*

We added the following sentences in M&M section, page 9:

“The random effect incorporated gender and age. To avoid a singular model fit resulting from the limited random effect, we defined the interaction between exposure score and BC grade. Moreover, we assumed that BC grade and our exposure score are interacted given their information source (i.e., histology appearance).”

*11. “All analyses were performed using 2,000-times bootstrap resampling“ I guess you meant you did analysis on the results of external validation set, right?*

*Authors’ reply:*

*Yes, it is. We accordingly added “external validation”.*

*12. Table 1, please add the criteria of malignancy grade for readership.*

Authors’ reply:

Added accordingly.

*13. Please use a consistent term of malignancy grade/cancer grade/tumor grade through the paper.*

Authors’ reply:

Changed accordingly.

*14. Please give an explanation of both subspace and space. The boundary definition of each subspace is also not clear to me. The t-SNE in Figure 3 didn’t show any clear subspace.*

Authors’ reply:

We agree with the reviewer that Figure 3 with 2D presentation is suboptimal to visualize 3D data. Accordingly, we provide as supplementary a video that navigates in the 3D t-SNE space.

In our study, we employed 3D t-SNE to analyze local features and identified global feature subspaces with a high concentration of patches dominated by either active smokers or non-smokers. These findings suggest a reorganization of histologic features associated with these subspaces. Our pathologists verified the presence of distinguishable histologic patterns within these subspaces using 3D t-SNE visualization. Importantly, the 3D visualization of feature spaces offers more comprehensive information compared to 2D visualization.

*We added the following paragraph in the discussion section, page 21:*

*“*In the current study, we further investigated the local features using 3D t-SNE and visualized the 3D feature spaces. We identified global feature subspaces that revealed a high concentration for patches dominated by either active smokers or non-smokers. These findings further indicate reorganization of histologic features associated with these subspaces. Our pathologists investigated and confirmed distinguishable histologic patterns between the subspaces we identified using 3D t-SNE visualization. We emphasize that the 3D visualization of feature spaces provides significantly more information than the 2D visualization of the feature spaces.”

*15. Number of patches of active smoker on in-training validation set was 3(0.09%). Why only 3 patches for this patient?*

Authors’ reply:

The epithelial components of the resection sample from this patient was sparse (~0.5 cm in diameter). We were subjected to select a model with more accurate prediction of patches originated from the never smoker cases (the in-training validation is a quasi-out-of-distribution set compared to the training set) as we assumed that the majority of histology appearance is driven by the common tumor biology and smoke exposure is an additional driver for histological alterations.

Accordingly, we added the following paragraph in the limitation section:

“In the in-training validation set, the resection sample from this patient exhibited sparse epithelial components, measuring approximately 0.5 cm in diameter. However, we prioritized the selection of a model that could provide more accurate predictions for patches derived from cases of individuals who had never smoked. It's important to note that the in-training validation set represents a quasi-out-of-distribution compared to the training set. Our rationale for focusing on never smoker cases was based on the assumption that the primary determinants of histology appearance are primarily related to common tumor biology, while smoke exposure acts as an additional factor driving histological alterations.”

*16. A typo in discussion. Auerbach(6) is in 1989.*

Authors’ reply:

Corrected accordingly.

*17. Supplementary Figure 1 need a figure caption.*

Authors’ reply:

Added accordingly.
