# Supplementary material for: Deep learning identifies histopathologic changes in bladder cancers associated with smoke exposure status
Source: PLoS One. 2024 Jul 31;19(7):e0305135. doi: 10.1371/journal.pone.0305135 (PMC11290674; doi:10.1371/journal.pone.0305135)
Supplement: Attachment — Submitted filename: review_comment_PLOSONE.docx [file pone.0305135.s008.docx]

**Thank you to the reviewers for your feedback. We hope our response is sophisticated. Changes in the main text were highlighted in yellow.**

**Response to Reviewer #1:**

1. **Inclusion of Former Smokers in the Training Cohort:** We recognize the reviewer's concerns about including former smokers in our training cohort. Adopting a category for former smokers as an indeterminate class is a recognized approach in machine learning. This practice introduces an indeterminate category which contributes to model regularization, a strategy aimed at preventing overfitting. Overfitting happens when a model is highly accurate on training data but performs poorly on new, unseen data. Our rationale for incorporating former smokers into the training dataset is based on the premise that their indeterminate histopathologic features and heterogenous patterns could enhance the model's ability to accurately distinguish between active smokers and never-smokers. This approach hopefully mitigates the model from learning indeterminate features found in the active smokers or never-smokers.

For testing, we opted to exclude former smokers due to the complexities involved in assessing tissue regeneration after smoking cessation. The inclusion of former smokers in our test analysis could potentially skew the results, considering their intermediate status blurs the lines between active and never smokers.

2. **Choice of Neural Architecture Search over Established Models:** Our research primarily focused on examining the relationship between smoking and histopathologic changes, rather than enhancing machine learning models. We employed NAS + PlexusNET, a well-examined neural network framework on across five medical image modalities by a previous study, comparing it to the Multiple Instance Learning (MIL) approach that reflects the global context learning. We also evaluated the models at 10x and 20x magnifications to understand the impact of magnification on histology-tobacco correlation.

While integrating models like ViT, ResNet, or CTransPath could have been beneficial, it was beyond our study's immediate scope. Previous research demonstrated that PlexusNET offers computational efficiency and competitive results in various medical imaging modalities. This indicates that our NAS-driven approach balances accuracy and efficiency better than traditional “established” models.

The Vision Transformer (ViT) model requires more computing power compared to PlexusNET and many typical CNN architectures. On the other hand, CTransPath uses CNN to detect local granular structures. Our PlexusNET, primarily a CNN model, is designed to identify fine granular structure associate to tobacco exposure. Additionally, PlexusNET can adopt a hybrid approach, combining CNN with Transformer, as optimized by Neural Architecture Search (NAS), for improved performance in capturing detailed patterns. Overall, in-depth discussion would be beyond the scope of the current study.

3. **Imbalanced In-Training Validation Set:** Our in-training validation set, which originated from a single center independent of both the training and test sets, exhibited a limited representation of active smokers but included a sufficient representation of former smokers. Our primary objective with this validation set is to emulate a one-class detection approach, aiming to identify a model that learned histopathologic features associated with individuals who have never smoked. Essentially, we are building a quasi-anomaly detection system, under the assumption that the underlying tumor biology is the primary factor influencing histological appearance. This approach allows us to evaluate the model's capability to identify histological changes linked to smoking as outlier.
4. **Clarification of Statistical Terms:** The variations in the confidence intervals for sensitivity and recall rates arise due to the application of bootstrapping methods with distinct seeds. This can lead to minor differences in the confidence intervals, even though the underlying measures remain the same. To avoid potential confusion for readers, we removed recall from the main text.
5. **Random vs. Fixed Effects in the Model:** In our analysis, we treated gender and age as random effects, recognizing them as demographic variables that were distributed randomly within the Prostate, Lung, Colorectal, and Ovarian (PLCO) Cancer Screening Trial cohort. This approach acknowledges the inherent variability in these factors across the population. Conversely, we designated time to diagnosis and malignancy grade as fixed effects. This decision aligns with the prospective nature of the PLCO study, where the time to diagnosis was meticulously recorded. We postulate that the time to diagnosis encompasses the latent period leading up to the clinical manifestation of bladder cancer. Furthermore, we suggest that the malignancy grade correlates with the smoking status of the individuals in the study. This correlation potentially extends to an indirect influence by the latent period and the duration of tobacco exposure within the time-to-diagnosis frame.
6. **Clarification on “Time to Diagnosis”:** The variable 'time to diagnosis' was derived from the PLCO study and represents the duration from the participants' inclusion in the screening study. We examined its correlation with smoking status, considering the length of exposure. Additionally, we assumed that 'time to diagnosis' might also be related to any latent period in the development of clinically visible bladder cancer, which could, in turn, influence the malignancy grade. This assumption is based on findings in the literature that suggest a connection between malignancy grade and smoking status.
7. **Explanation of Mixed Effect Regression Model Symbols:** We have added clarification in our manuscript regarding the symbols used in the mixed-effect regression model. The symbols represent interaction (.), conditional relations (||), and grouping (()). These are standard notations in statistical modeling.

**Response to Reviewer #3:**

1. **Classification Performance and Reliability:** We appreciate the reviewer's concerns regarding the AUC of 0.67. However, it's essential to provide clarity on the primary objective of our study, which was to explore the association between histology and smoking status, rather than solely aiming for the highest classification performance.

We would like to reiterate, as mentioned in our initial revision letter, that our approach is in alignment with standard statistical practices. It's important to understand that modeling can serve two primary purposes: investigating associations or making predictions (e.g. classification). In our study, we focused on examining associations rather than predictions, and the study design was accordingly tailored to this objective.

We would like to highlight the robust statistical power of our study, with a confidence level of 82.2% at a 5% alpha level. This statistical strength reinforces the credibility and reliability of our findings, especially within the specific scope of our research objectives.

Moreover, we firmly believe that our assessment is both objective and well-founded. It aligns with a prior study (cited in the main text) that also hinted at an association between tobacco exposure and histopathological changes that were not considered for malignancy grading. Additionally, multiple other studies have indicated the existence of a potential relationship between malignancy grades and tobacco exposure in bladder cancer (already referenced in the main text).

In consideration of these compelling factors, we firmly believe that our study contributes valuable insights to the field. It sheds light on the intriguing connection between histopathology and tobacco exposure, which we anticipate will ignite further evaluation of the impact of environmental or human factors on cancer histology, opening new avenues for research and understanding in this domain.

2. **Rationale for Including Former Smokers:** Including a class for former smokers as indeterminate or heterogenous class in a machine learning model is a recognized practice. This approach introduces an indeterminate category that can aid in the regularization of the model. Regularization, in machine learning, is a technique used to prevent overfitting, where the model performs well on the training data but poorly on new, unseen data. The rationale is based on our assumption that their indeterminate histopathologic characteristics or heterogenous patterns might facilitate our models to learn most distinguishable patterns for the active smokers and never-smokers. This differentiation can provide more nuanced insights and improve the model's ability to generalize for never-smoker and active smokers.

Ablation studies are useful for understanding the impact of certain features or categories on the model's performance, but they can also be resource-intensive and does not align with the specific goal of our study which is to investigate the association between histology images and smoke exposure.

Our approach, focusing on a training set that includes a broad spectrum of cases (including former smokers) and a highly curated test set, is aimed at ensuring that the model is capable to detect association between tobacco exposure (active vs never) and histology. This methodology is consistent with best practices in machine learning, where diverse training data helps in building robust models, and a well-curated test set is crucial for accurately assessment.

3. **Consistency in Testing and Training:** The use of a two-class system in our test set (active vs. never smokers) was intentional, aligning with our research objectives and already justified in the previous points. The threshold of 65 was determined using the median value on the development set and fixed for the test set, which is a standard approach in such analyses. We highlighted the following sentence related to thresholding (highlighted in green in the main text, page 8).

“Afterward, the median exposure score was estimated from the development data set and used as the threshold for conversion of the exposure score from a continuous to binarily categorized parameter, and this was locked as the threshold for the external validation set.”

4. **Optimal Magnification Selection:** While comprehensive investigations across different magnifications might be informative, our study did include the investigation on 10x and 20x objective magnification. Model resulting from NAS was identified on development set with 32x32 pixels downsized images following the instruction published earlier, which we think to be reliable for our study’s purpose, minimizes the impact of magnification on the model selection. We refer the reviewer to our previous work that thoroughly investigated our NAS strategy on 5 different medical imaging modalities including pathology images.

In summary, NAS was not utilized for either 10x or 20x magnification, rendering the concerns unfounded.

5. **3D t-SNE Visualization and Feature Representation:** The 3D t-SNE visualization serves as a tool for data exploration and facilitates the understanding of feature representation by visualizing them in a manner that is browsable and readable for humans to identify subspaces relevant for the study question. We labelled the data points according to the smoke status and exposure score to visually identify the subspaces we described in the main text as well while ensuring a sophisticated case coverage in these subspaces. Our pathologists have confirmed the distinct histologic patterns observed in the identified feature subspaces we described in the main text, and we provided multiple images originated from these subspaces to validate our perspective in the supplementary material section. We refer the reviewer for further information regarding the t-SNE visualization as it is beyond the scope of the current study to investigate the limitations of t-SNE or to provide alternative solutions.
6. **Justification of K-fold Cross-Validation Setting:** Our decision to use K=2 in cross-validation during NAS was based on previously established rationales in our earlier work which the computational efficacy and the completion of NAS within 24 hours using a single GPU. We showed that our NAS approach using 2-fold CV deliver models that are on par and superior to existing SOTA models. Moreover, expanding on this is beyond the current study’s scope. Accordingly, we refer the reviewer to our earlier work (<https://pubmed.ncbi.nlm.nih.gov/36753979/>) that justified the use of the 2-fold cross validation.
