# Supplementary material for: Deep learning identifies histopathologic changes in bladder cancers associated with smoke exposure status
Source: PLoS One. 2024 Jul 31;19(7):e0305135. doi: 10.1371/journal.pone.0305135 (PMC11290674; doi:10.1371/journal.pone.0305135)
Supplement: Attachment — Submitted filename: review_comment_PLOSONE_last.docx [file pone.0305135.s009.docx]

We thank the reviewer for the valuable feedback.

Reviewer #1:

The reserch is intresting and it is maybe decover the relationship between the smoking and the histopathologic changes.

**Authors’ reply:**

**We appreciate the positive feedback and recognition of the relevance of our work.**

Only the result of deep learning model is given, the result of baseline method is absent.

**Authors’ reply:**

**Given that Grade is not a significant independent predictor of smoke exposure in the** **multivariate mixed-effect logistic regression, there is no point in providing the performance in AUC (as a measurement of randomness) or F1 score (as a measurement of accuracy) of the baseline method. We already provided MIL performance as a comparison on page 16, line 4:**

**“In addition, MIL failed to provide a non-random prediction for smoke exposure status (AUROC: 0.561; 95% CI: 0.437 – 0.685).” Accordingly, given the randomness of prediction for MIL, there was no point in providing an F1 score here.**

The details of the model is necessary, such as a charflow or a network frame diagram.

**Authors’ reply:**

**We provided a model architecture configuration following definitions published in the previous paper (https://pubmed.ncbi.nlm.nih.gov/36753979/). Additionally, we included the PlexusNET general network concept as supplementary Figure 3.**
